# PePPER: a webserver for prediction of prokaryote promoter elements and regulons

**DOI:** 10.1186/1471-2164-13-299

**Published:** 2012-07-02

**Authors:** Anne de Jong, Hilco Pietersma, Martijn Cordes, Oscar P Kuipers, Jan Kok

**Affiliations:** 1 Department of Molecular Genetics, University of Groningen, Groningen Biomolecular Sciences and Biotechnology Institute, 9747 AG, Groningen, The Netherlands; 2 Top Institute Food and Nutrition, Wageningen, The Netherlands; 3 The Netherlands Kluyver Centre for Genomics of Industrial Fermentations, Delft, The Netherlands/Netherlands Consortium of Systems Biology, Amsterdam, The Netherlands

## Abstract

**Background:**

Accurate prediction of DNA motifs that are targets of RNA polymerases, sigma factors and transcription factors (TFs) in prokaryotes is a difficult mission mainly due to as yet undiscovered features in DNA sequences or structures in promoter regions. Improved prediction and comparison algorithms are currently available for identifying transcription factor binding sites (TFBSs) and their accompanying TFs and regulon members.

**Results:**

We here extend the current databases of TFs, TFBSs and regulons with our knowledge on *Lactococcus lactis* and developed a webserver for prediction, mining and visualization of prokaryote promoter elements and regulons via a novel concept. This new approach includes an all-in-one method of data mining for TFs, TFBSs, promoters, and regulons for any bacterial genome via a user-friendly webserver. We demonstrate the power of this method by mining WalRK regulons in Lactococci and Streptococci and, vice versa, use *L. lactis* regulon data (CodY) to mine closely related species.

**Conclusions:**

The PePPER webserver offers, besides the all-in-one analysis method, a toolbox for mining for regulons, promoters and TFBSs and accommodates a new *L. lactis* regulon database in addition to already existing regulon data. Identification of putative regulons and full annotation of intergenic regions in any bacterial genome on the basis of existing knowledge on a related organism can now be performed by biologists and it can be done for a wide range of regulons. On the basis of the PePPER output, biologist can design experiments to further verify the existence and extent of the proposed regulons. The PePPER webserver is freely accessible at http://pepper.molgenrug.nl.

## Background

As early as in 1960 the term operon was coined for a group of genes of which the expression is coordinated by an operator [1]. Experimental methods like Electrophoretic Mobility Shift Assays (EMSA), Surface Plasmon Resonance (SPR), nuclease protection assays (DNAse-footprinting) and Chromatin Immuno Precipitation (ChIP) can all be used to demonstrate that an interaction exists between a transcription factor (TF) and DNA [2]. Experimentally proven TFBSs have been described in literature and are available via publicly accessible databases such as DBTBS [3], RegulonDB [4], PRODORIC [5], MicrobesOnline [6], RegTransBase [7] and RegPrecise [8]. Besides experimental proof for the existence of protein-DNA interaction, TFBS discovery algorithms have been developed to uncover conserved regions that might act as TFBSs (MEME [9], ARCS-Motif [10], GLAM2 [9], W-AlignACE [11], GIMSAN [12], RankMotif++ [13], GAME [14], and Tmod [15]). This so-called motif mining is based on a collection of genes having a certain correlation. Gene-to-gene correlations can be derived e.g., from transcriptome data or from functional relations like belonging to the same metabolic pathway or to certain COG or GO classes. Motif mining consists of a search for conserved DNA patterns in the upstream intergenic regions of the genes or the operons to which the gene(s) belong. A high probability (low *p*-value) that the occurrence of a certain DNA pattern is very specific for a gene set does not necessarily imply that this motif constitutes a TFBS but it is a good lead for biological functional analysis.

### Regulons

Genes and operons that are under control of the same TF are members of that TF’s regulon. Although methods for the prediction of regulons have been substantially improved [16], they are still far from perfect. Comparative genomics tools can be used to predict regulons in bacterial genomes but the procedure can lead to incorrect regulon calling. Despite this drawback, several regulon databases are available that are based on comparative genomics methods and lack experimental evidence. Probably the most extended and accurate databases of regulons are DBTBS for *B. subtilis*[3] and RegulonDB for *E. coli*[4]. The latest update of DBTBS brought the total number of *B. subtilis* TFs to 120, promoters to 1475 and regulated operons to 736, of which 463 operons have been experimentally validated [3]. Together, RegulonDB and DBTBS are the major resources for regulon network mining dedicated to prokaryotes. PRODORIC and RegTransBase are the most extended and manually curated databases on gene regulation in prokaryotes in general [5]. Besides regulon information they include TFBSs and bioinformatics tools for prediction, analysis and visualization of gene regulatory networks using ProdoNet [17] and furthermore, PRODORIC offers the tool “virtual footprint”, which can be used to mine for novel regulons. The *in silico* prediction of regulons is usually based on operons that share the same TFBS and the information is supplemented with the results from comparative genomics analysis of known regulons. This method is used in the recently launched webserver RegPrecise [8], which gives access to a database containing a collection of manually curated regulons grouped together by similar properties such as belonging to the same biological process or metabolic pathway. The database is limited to six closely related bacteria (*Shewanella, ThermotogalesBacillales* and *Desulfovibrionales*). On the other hand FITBAR [16] is dedicated to TFBS mining and discovery, RegAnalyst [18] and ProdoNet [17] are webservers enabling integration of data on proteomics and metabolic pathways and provide subsequent graphical representation of networks.

In this work, we designed and developed a novel tool, PePPER, to mine for regulons and TFBSs in any sequenced bacterial genome. As a showcase, we extended the existing regulon databases with a database of *L. lactis* regulons that is derived from literature on transcriptional regulation. The latter is accessible via the user-friendly PePPER web interface.

## Implementation

### Data resources

MolgenRegDB is an integrated *in house* collection of TFs, TFBSs and regulons of *L. lactis* and is available via the PePPER webserver (http://pepper.molgenrug.nl). In addition, TF and TFBS data were downloaded from RegulonDB (*E. coli*) and DBTBS (*B. subtilis*) and subsequently reformatted and integrated together with MolgenRegDB in the PePPER database. Data of all publically available bacterial genomes are daily updated from NCBI (http://www.ncbi.nlm.nih.gov) and available via the PePPER webserver.

### Implemented mining tools

Overrepresented DNA motifs are identified using MEME [9] and the position-specific probability matrices (PSPMs) obtained were converted to position weight matrices (PWMs) that are compatible with MOODS [19]. BLAST 2.2 [20] is used for protein comparisons. Glimmer3 [21] is used for automated gene detection (open reading frame or ORF calling) and Ribosomal Binding Sites (RBSs) are detected using RBSfinder [22]. In case of *de novo* ORF calling, the translation start is adapted to match the RBS prediction, otherwise the original annotation is used. TransTermHP [23] is implemented for the discovery of putative transcription terminators. Possible secondary RNA structures are predicted and plotted using RNAfold and RNAplot of the Vienna package [24]. A new prokaryote promoter prediction tool was developed and is based on PWMs and Hidden Markov Models (HMMs) of −35 and −10 consensus sequences and various sigma factor binding sites. PWMs and HMMs of *B. subtilis* and *E. coli* promoters are used as reference for Gram-positive and Gram-negative bacteria, respectively. A collection of individual tools used by PePPER are accessible via the webserver.

### Building the *L. Lactis* TFBS library

A database of validated *L. lactis* TFBSs of regulons derived from literature data was made after which for each TFBS a PSPM was calculated using MEME and subsequently transposed to a MOODS compatible PWM format. To that end we used the upstream intergenic regions plus the first 20 bases of their genes as input for MEME in order to search for overrepresented DNA motifs. These motifs ranged in length from 6 to 18 bases and a database of all intergenic regions of *L. lactis* MG1363 was used as a background model. Subsequently, the overrepresented DNA motifs were manually compared to the literature data. Only those DNA motifs that resemble the experimentally verified TFBSs were included in the database, including the MOODS cutoff values. An overview of TFBSs of regulons, including WebLogos, is shown in Additional file 1: Table S 1; the database containing all the PSPM profiles is available via the PePPER webserver.

### The PePPER toolbox

A powerful toolbox has been created in PePPER and is accessible via the PePPER webserver. By selecting a regulon on the basis of its TF and one or more genomes, the program will perform a Blast analysis between the proteins of the known regulon and all the proteins encoded by the genes in the selected genome(s). PePPER provides a clear overview in colors, of the predicted regulon(s), in each genome, which is based on the degree of protein homologies; detailed information is given in attached tables. More details about scoring and the color scheme are given on the PePPER webserver.

### PePPER all-in-one

**Figure 1:**
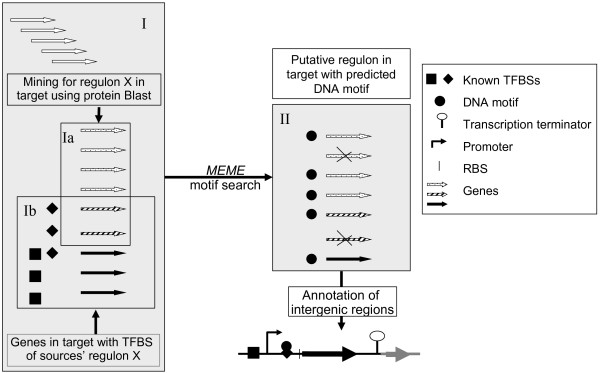
**Flow diagram of PePPER all-in-one.** The first step of PePPER all-in-one is to select a set of genes that putatively belong to a certain regulon in one organism (target; Box I represents all genes of the target organism) through comparison with the corresponding regulons in all other organisms (source) using protein Blast (genes in Box Ia). In parallel, the known TFBSs of these regulons are used to find genes in the target organism that carry this DNA motif in their upstream regions (genes in Box Ib). Subsequently, a MEME search is performed on the upstream regions of the genes in both independently obtained gene pools. This results in a set of genes that represents the putative regulon in the target organism with its predicted TFBS (genes in Box II). Finally, features such as RBSs, promoter and transcription terminators are added, after which the result is graphically represented. The information can be accessed and viewed separately per gene.

PePPER all-in-one is a parameter-free pipeline of the individual PePPER tools allowing fully automatic intergenic annotation combined with analysis of regulons. A schematic overview of the PePPER all-in-one process is shown in Figure 1. Two input formats are accepted for analysis: i) plain DNA sequences in FastA format, ii) a fully annotated file in the Genbank file format. DNA sequences lacking ORF information will be automatically annotated using Glimmer3 to discriminate between ORFs and intergenic regions. Input files in Genbank format, either uploaded or selected from the PePPER library of genomes, will produce the most extended results, including hyperlinks to NCBI resources such as protein annotation, protein domains and genomic context of the genes. The output is organized into three tables and one figure: i) Table 1, the “Summary of Results” contains links to detailed information on analysis of regulons, TFBS, promoters, transcription terminators, RNA folding and motif analysis using MEME, ii) Table 2 and Table 3, “Files available for download”, iii) Table 4, Combined results of the TFBS and regulon mining. Figure 1 gives a graphical presentation of the intergenic regions.

**Table 1:** **Regulators of which the regulons have been studied in** *** Lactococcus lactis* ssp.** *** cremoris* MG1363 and** *** Lactococcus lactis* spp.** *** lactis* IL1403 and their literature references. -, strain/subspecies not specified**

**Gene**	**Literature**
AhrC	MG1363 [25,26]
ArgR	MG1363 [25,26]
BusR	-[27,28]
CcpA	MG1363 [29]
CodY	MG1363 [30,31]
ComX	-[32]
CopR	IL1403 [33]
CtsR	MG1363 [34,35]
FhuR	IL1403 [36]
FlpA	MG1363 [37]
FlpB	MG1363 [37]
FruR	-[38]
GadR	-[39,40]
GntR	MG1363 [41]
HdiR	MG1363 [42]
HisZ	-[43,44]
LlrA	MG1363 [45]
LlrB	MG1363 [45]
LlrC	MG1363 [45]
LlrD	-[46]
LlrE	MG1363 [45]
LlrF	MG1363 [45]
LlrG	MG1363 [45]
LmrR	MG1363 [47,48]
MalR	-[49]
PhoU	-[50]
PurR	-[51,52]
PyrR	MG1363 [53]
RcfB	-[54]
SpxA	-[55,56]
XylR	-[57]
ZitR	MG1363 [58]

**Table 2:** Analysis of regulons

***B. subt***	***L.lactis*** **MG1363**				**Blast**
	**locus tag**	**gene name**	**Protein ID**	**GeneID**	**e-value**
*walK*	llmg_0414	*llrC*	YP_001031764.1	4797664	2.00E-91
*walR*	llmg_0413	*kinC*	YP_001031763.1	4798420	1.00E-95
*yycJ*	llmg_0412	*vicX*	YP_001031762.1	4798732	4.00E-78
*yycK*	llmg_2419	*htrA*	YP_001033660.1	4797497	3.00E-74
*ftsA*	llmg_2061	*ftsA*	YP_001033316.1	4797264	3.00E-74
*ftsZ*	llmg_2060	*ftsZ*	YP_001033315.1	4798073	5.00E-106
*phoP*	llmg_0414	*llrC*	YP_001031764.1	4797664	2.00E-67
*phoR*	llmg_0413	*kinC*	YP_001031763.1	4798420	2.00E-51
*tagB*	llmg_1603	*tagB*	YP_001032887.1	4798977	1.00E-32
*tagD*	llmg_1606	*tagD2*	YP_001032890.1	4798976	3.00E-33
*tagF*	llmg_1604	*tagF*	YP_001032888.1	4798736	2.00E-73
*yjeA*	llmg_0293	*xynD*	YP_001031648.1	4797603	3.00E-43
*yocH*	llmg_2194	*llmg_2194*	YP_001033444.1	4798120	2.00E-22

**A.** Comparison of the *B. subtilis* WalRK regulon to the *L. lactis* MG1363 genome.

**Table 3:** **Comparison of the WalRK TCS of** *** B. subtilis* to the** *** L. lactis* orthologs using PePPER’s multiple genome regulon mining tool**

***B. subtilis***	***L. lactis***			
	**MG1363**	**IL1403**	**SK11**	**KF147**
*walR*	*llrC*	*llrC*	*LACR_0444*	*tcsR*
*walK*	*kinC*	*kinC*	*LACR_0443*	*tcsK*
*yycJ*	*vicX*	*yeaA*	*LACR_0441*	*yeaA*
*yycK*	*htrA*	*htrA*	*LACR_2439*	*htrA*

**B**. Note that a nomenclature mix up took place in the *B. subtilis* 168 NC_000964 file (release Feb. 2011); in this release, YycG (locus BSU40400**)** is called WalK, but it is described as “two-component sensor histidine kinase YycF” while YycF is labeled WalR “two-component response regulator YycG”. The names WalR and WalK have recently been corrected. Due to this temporary swap, the annotation of *yycF* and *yycG* could still be wrong in the annotation of other bacterial genomes.

### Promoter prediction using PePPER

A universal prokaryote transcription initiation DNA motif does not exist [2], but a common DNA pattern (the Pribnow box) 10 base pairs upstream of the transcription start site (TSS) and a conserved sequence 35 base pairs upstream of the TSS are overrepresented in promoter regions. These patterns are searched for separately, after which putative promoters are only taken into account if the spacing between their −35 and −10 motifs is 16 to 18 bases. Although many different sigma factors binding sites are known (especially from *B. subtilis*) these are not used in the promoter prediction routine used here; they are implemented as conserved DNA motifs in the TFBS mining tool. The resulting promoter prediction algorithm is universal for prokaryotes, but we do offer the possibility to discriminate between Gram-positive and Gram-negative bacteria to improve the accuracy of the prediction algorithm. Furthermore, “incomplete” promoters, in which only a −35 or a −10 sequence is predicted are also shown in the results.

### Webserver

PePPER (http://pepper.molgenrug.nl) can be accessed through a user-friendly web interface for querying and browsing. The server runs on a linux platform (Ubuntu server LTS 10.04) with an Apache webserver (version 2.2) and a MySQL server (version 5.1) and Blast 2.2. Programming was done using PHP 5.0, Perl 5.12 and BioPerl 1.8. A combination of Joomla and jQuery 1.4 was used to build the user-friendly web interface.

## Results and discussion

### Regulons in *lactococcus lactis*

Each of the 154 known or predicted TFs of *L. lactis* subsp. *cremoris* MG1363 [59] will probably regulate the transcription of one or more genes or operons. The functionality of 32 TFs of *L. lactis* MG1363 and *L. lactis* subsp. *lactis* IL1403 has been reported in literature, using techniques ranging from DNA microarray analysis to DNA footprinting. Although the two lactococcal subspecies are closely related, not each regulator or regulon of one is present or similar in the other. The majority of the TFs in MG1363 and IL1403 show a high degree of mutual similarity. Of the 154 TFs in *L. lactis* MG1363, 22 are not present in *L. lactis* IL1403 while 20 out of the 143 TFs identified in *L. lactis* IL1403 are not found in MG1363 (Tables 2 and Table 3). Analysis performed by PePPER showed that large regulons (those of CodY, CcpA, CmbR, CesSR, ArgR, and PurR) as well as some small regulons (those of RcfB, ZirR, BusR and LmrR) are well conserved in the two strains. The conservation of regulons between the closely related subspecies is illustrated by the CmbR regulon of cysteine and methionine biosynthesis, which has been studied in detail in both *L. lactis* IL1403 [60] and *L. lactis* MG1363 [36]. Analysis of both CmbR regulons shows that 16 out of 17 proteins in the IL1403 CmbR regulon have high similarity to MG1363 proteins (data not shown). Finally, all known TFs and TFBSs of *L. lactis* were collected in one database, the MolgenRegDB. This is currently the most comprehensive manually curated regulon database of *L. lactis*; it is available via the PePPER webserver (http://pepper.molgenrug.nl).

### Prediction of the WalR regulon in four *L. Lactis* strains

The *B. subtilis* operon *walRKyycHIJK* is a 6-cistron operon encoding among others the two-component system (TCS) WalRK that controls the expression of 23 genes. These genes represent the WalR regulon [61-64]. This signal transduction pathway is crucial for the regulation of cell wall metabolism and is one the few TCS known to be a virulence factor in *S. pneumoniae*[61]. The presence of the WalR regulon has never been described in *L. lactis*. We validated PePPER by comparing its results to literature data and subsequently used it to unravel the putative WalR regulons and cognate TFBS in 4 sequenced strains of *L. lactis*. PePPER showed that the products of 4 of the genes of the *walRKyycHIJK* of *B. subtilis* are orthologous to *kinC, llrC* and *vicXhtrA* of *L. lactis* MG1363 (see Table 3). Furthermore, PePPER showed that 13 out of the 23 proteins of the WalR regulon of *B. subtilis* show high similarity (Blast e-value <10^−20^) to proteins in *L. lactis* MG1363; they are organized in 6 operons (Table 2). Using PePPER’s multiple genome mining tool, it is clear that orthologs of the WalRK TCS and part of the WalR regulon genes of *B. subtilis* are present in all other fully sequenced *L. lactis* strains: IL1403, SK11 and KF147 (Table 3).

**Figure 2:**
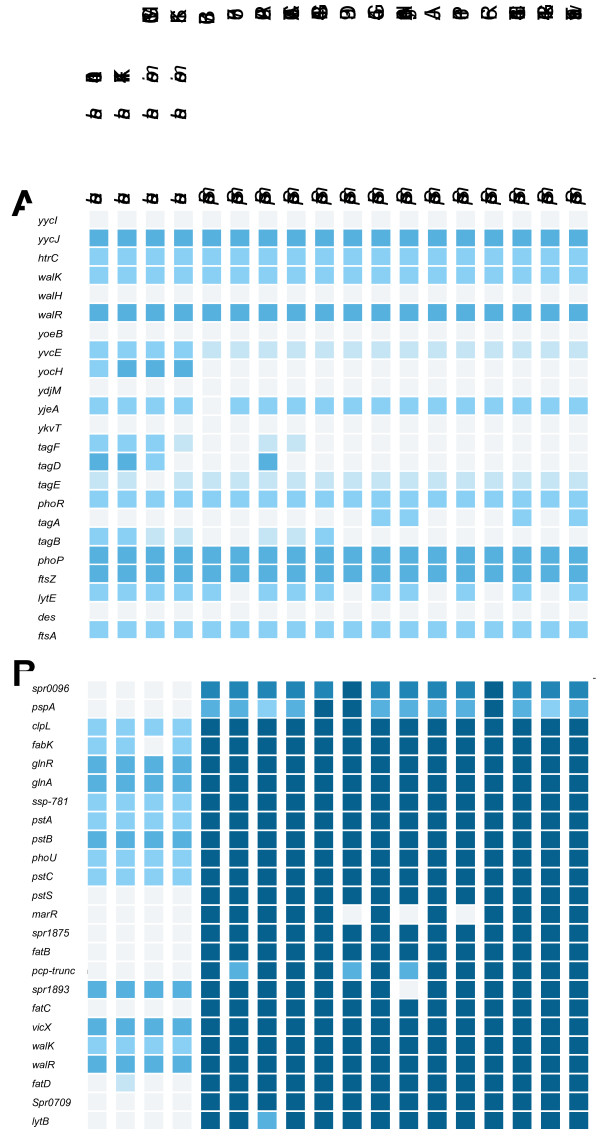
**Presence of regulon in query organisms by protein Blast.** WalRK regulon genes of *B. subtilis* (**a**) and *S. pneumoniae* (**b**) were used to perform a protein Blast in order to examine whether a WalRK regulon might be present in Lactococci and Streptococci. Colors from light to dark blue are indicative of increasing protein similarity; for more details see the PePPER webserver.

Streptococci are phylogenetically closely related to Lactococci and therefore we performed an analysis of the WalRK regulons in both groups using the PePPER toolbox. The result (Figure 2) shows the similarity of the known WalRK regulons (*B. subtilis, S. pneumoniae, Staphylococcus aureus*) with the predicted regulons of Lactococci and Streptococci. The *B. subtilis* regulon (Figure 2a) showed the high conservation between the two groups where 6 genes were not found and 9 genes out of 23 were found in all species (protein blast e-value cutoff of 10^−20^). The difference between the two groups is shown in Figure 2b where 11 out of 24 genes of the *S. pneumoniae* R6 WalRK regulon were not found in Lactococci.

### TFBS prediction

**Figure 3:**
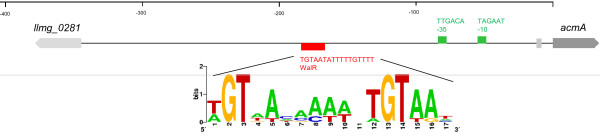
**Annotation of the intergenic region upstream of *acmA*.** The genes *acmA* and the first upstream gene, *llmg_0281*, are indicated with gray arrows and are not drawn to scale. The WebLogo is based on the MEME weight matrix; light grey box is the predicted RBS. No transcription terminator was found in this area.

The WalR binding site (TGTAA-n6-TGTAA) was mapped using DNAseI footprinting and EMSAs in 4 organisms; *B. subtilis, Staph. aureus, S. pneumoniae* and *S. mutans*[61]. We added the WalR TFBSs derived from these 4 organisms separately, as well as an averaged (combined) WalR motif (WalR_[combined]_) to the PePPER database and subsequently screened the genomes of the *L. lactis* strains for the presence of these DNA motifs. The upstream DNA regions of two genes of *L. lactis* MG1363 that are orthologous to WalRK regulon members carry the WalR_[combined]_ TFBS, namely *xynD* (TGTAT-n6-TGTTA) and *htrA* (TGAAA-n6-TGAAG). In the upstream DNA region of the other 4 WalRK operon orthologs no WalR_[combined]_ was found. Interestingly, WalR_[combined]_ (Figure 3) was present upstream of the cell wall hydrolase genes *acmA* and *acmB*[65,66], which could indicate that these genes might be regulated by WalRK and that the WalRK stress response system of *L. lactis* also influences their expression. 

### PePPER all-in-one case study

**Figure 4:**
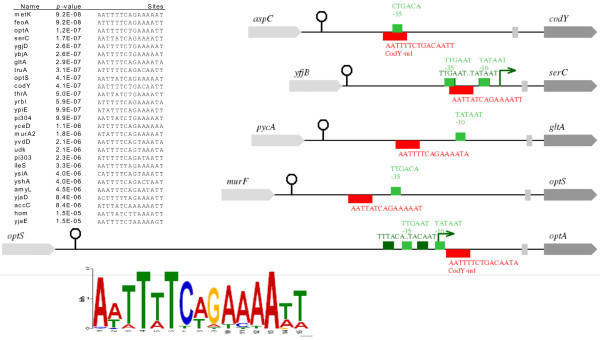
**CodY regulon prediction by PePPER all-in-one.** The inserted table shows the CodY-TFBS_[MG1363]_ found in *L. lactis* IL1403 (*p*-value < 10^-5^); the drawings show the intergenic regions upstream of CodY regulon members. At the bottom of this figure the WebLogo is shown of the TFBS derived from the MEME search. Predicted promoters with a correct spacing between −35 and −10 are colored dark green. Green arrows indicate predicted TSSs, light green boxes represent individual conserved −10 and −35 DNA motifs.

The well-studied global transcriptional regulator CodY of *L. lactis* MG1363 [67,68] binds to the CodY-box (AATTTTCWGAAAATT) and influences the expression of genes involved in (branched-chain) amino acid uptake and biosynthesis as well as several other genes involved in N-metabolism (proteolysis and peptide uptake). The CodY regulon of *L. lactis* MG1363 was used in the PePPER all-in-one system to mine the *L. lactis* IL1403 genome for the presence of homologs of the CodY_[MG1363]_ regulon and the CodY-TFBS_[MG1363]_. Subsequently, a MEME search was performed. The results showed that a CodY-TFBS is present in the upstream intergenic regions of 5 genes/operons in *L. lactis* IL1403, namely *codY, serCAB, gltA-citB-icd, dppA* and *dppPBCDF*. In *L. lactis* IL1403, Dpp, erroneously annotated as Opt [69], functions as a di/tripeptide transporter, with DppA as the substrate binding protein, and as an oligopeptide transporter employing DppP. The *dppP* gene in the *dppA-dppPBCDF* gene cluster of *L. lactis* MG1363 is mutated; in this strain CodY binds upstream of *dppA* but not upstream of *dppP*. Oligopeptide uptake in *L. lactis* MG1363 is encoded by the *oppDFBCA* operon, which is under CodY control [68]. The Opp system in *L. lactis* IL1403 is present but non-functional [69] and no CodY-TFBS_[MG1363]_ was found in the DNA region upstream of *opp*. Despite the differences in the activities of these transport systems, our analysis indicates that in both lactococcal strains CodY regulates di/tri- and oligopeptide transport. The graphical overview of intergenic regions (Figure 4) shows that CodY represses gene expression by binding in or closely downstream of the promoter regions. The DNA binding motif that was identified in *L. lactis* IL1403 resembles CodY-TFBS_[MG1363]_[68] and the CodY-TFBS_[IL1403]_ reported by Guedon et al. [67]. 

We used the CodY_[MG1363]_ regulon to screen for the presence of a similar regulon in a less closely related Gram-positive bacterium, the pathogen *S. pneumoniae* D39. The analysis revealed that seven genes/operons (*ilvD, ilvE, asd, hom-thrB, amiACDEF, SPD_1878-thrC, livJHMGF*) involved in amino acid transport or biosynthesis carry a sequence closely related to CodY-TFBS_[MG1363]_ in their upstream DNA regions.

## Conclusions

PePPER uses a novel approach, in which all available information on prokaryotic regulons and TFBSs is used to identify regulons in any query bacterium. In addition it offers a user-friendly web interface making the data provided byPePPER easily accessible for non-bioinformaticians. PePPER offers, next to all fully sequenced bacterial genomes, the possibility to upload un-annotated data, which is then processed automatically. Furthermore, prediction of intergenic region elements such as promoters, transcription terminators, sigma factor binding sites, RBSs, as well as that of possible secondary DNA structures therein, will lead to more detailed knowledge of the DNA regions under study. By adding our knowledge on *L. lactis* regulons as well as DBTBS and RegulonDB regulon data to the PePPER database, we provide an extended database of bacterial regulons and TFBSs. PePPER can be used to pinpoint a wide range of putative regulons and their cognate TFBSs in any bacterial genome on the basis of existing knowledge. This regulon information can subsequently be used by biologists to help them design experiments to authenticate the proposed regulons.

## Competing interests

The authors declare that they have no competing interests.

## Authors’ contributions

AJ and JK devised the PePPER concept and web design and wrote the manuscript. OPK participated in the design of the study and helped to draft the manuscript. HP and MC contributed to the writing of the webserver scripts and to building of the PePPER database. All authors read and approved the final manuscript.

## Supplementary Material

Additional file 1 Table S1 *Lactococcus lactis* TFBS WebLogos. Column 1 presents all known (studied) regulons of *L. lactis*. Alternative names for TFBS are given in column 2. The consensus sequence given in columns 3 were taken from the literature references from Table 1. In column 4, the TFBS identities are given that are used by PePPER. The upstream sequences of the genes of the regulons indicated in column 1 were aligned using MEME [9]. The obtained DNA motifs (WebLogos) are presented in column 5. Click here for file
