# Supplementary material for: PePPER: a webserver for prediction of prokaryote promoter elements and regulons
Source: BMC Genomics. 2012 Jul 2;13:299. doi: 10.1186/1471-2164-13-299 (PMC3472324; doi:10.1186/1471-2164-13-299)
Supplement: Additional file 1 Table S1 — Lactococcus lactis TFBS WebLogos. Column 1 presents all known (studied) regulons of L. lactis. Alternative names for TFBS are given in column 2. The consensus sequence given in columns 3 were taken from the literature references from Table 1. In column 4, the TFBS identities are given that are used by PePPER. The upstream sequences of the genes of the regulons indicated in column 1 were aligned using MEME [9]. The obtained DNA motifs (WebLogos) are presented in column 5. [file 1471-2164-13-299-S1.pptx]

## Slide 1
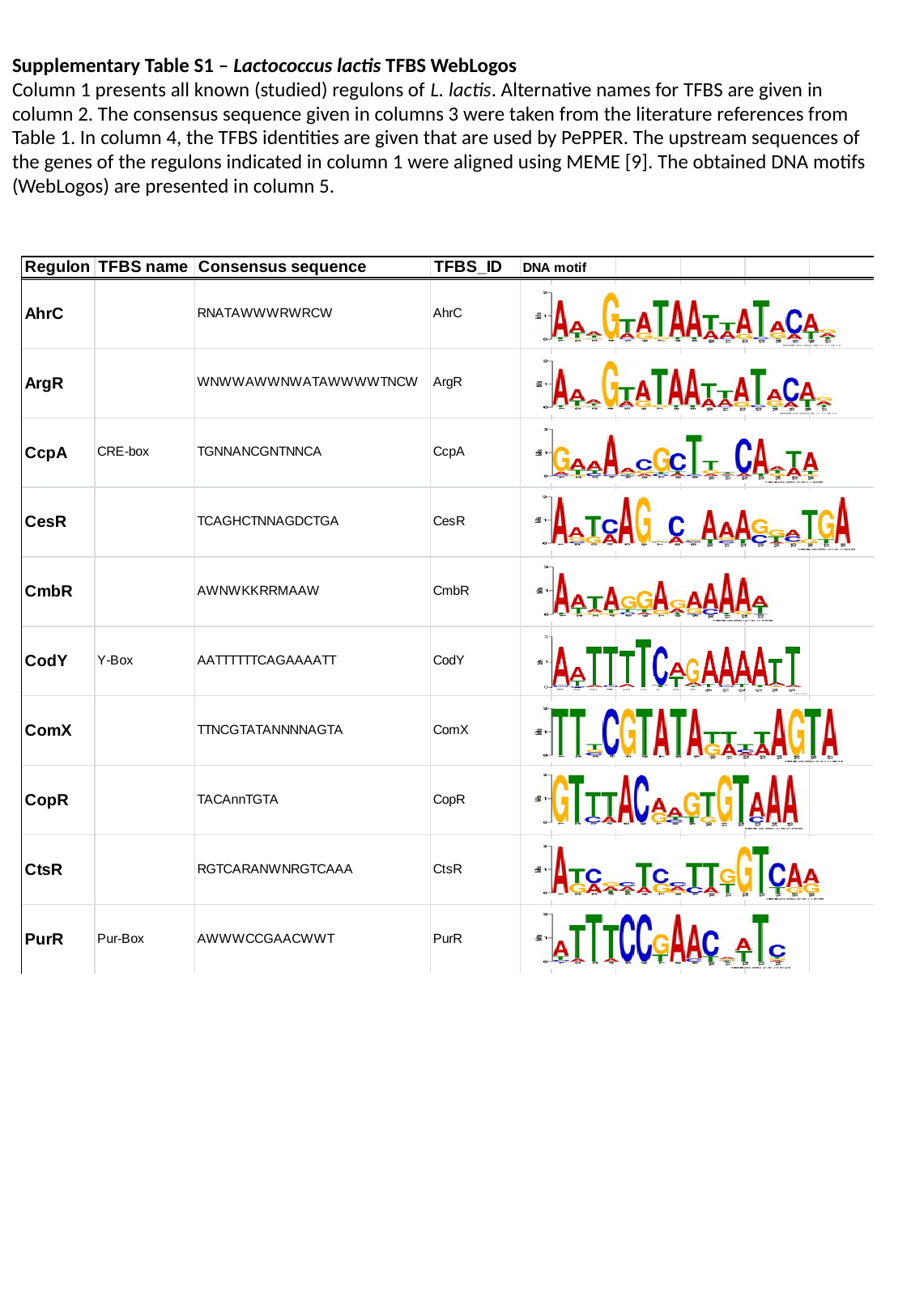

Supplementary Table S1 – Lactococcus lactis TFBS WebLogos
Column 1 presents all known (studied) regulons of L. lactis. Alternative names for TFBS are given in column 2. The consensus sequence given in columns 3 were taken from the literature references from Table 1. In column 4, the TFBS identities are given that are used by PePPER. The upstream sequences of the genes of the regulons indicated in column 1 were aligned using MEME [9]. The obtained DNA motifs (WebLogos) are presented in column 5.
